# Two Unusual Mutations in the Epidermal Growth Factor Receptor Gene in a Patient With Lung Adenocarcinoma

**DOI:** 10.7759/cureus.22372

**Published:** 2022-02-18

**Authors:** Martin Zapata Laguado, Andrea Zuluaga, Rafael Parra Medina, Ricardo Bruges

**Affiliations:** 1 Clinical Oncology, Instituto Nacional de Cancerología, Bogotá, COL; 2 Pathology, Instituto Nacional de Cancerología, Bogotá, COL

**Keywords:** egfr gene, egfr mutations, lung adenocarcinoma, non-small-cell lung, osimertinib

## Abstract

Adenocarcinoma (ADC) of the lung is the most frequent pathology corresponding to non-small cell lung cancer (NSCLC). The advent of target therapy and the discovery of drugs that block signaling pathways related to cellular events involved in the progression of the disease have led to a better prognosis in cases of ADC. Some of the targeted therapy focuses on the blockade of epidermal growth factor receptor (EGFR), targeting mutations in exon 19 and 21, with favorable clinical outcomes. However, there is limited evidence with respect to unusual mutations as in exon 18 (g719x) and 20 (s768). In this report, we present a case of a 65-year-old female with two unusual mutations in the EGFR gene, in exon 18 (g719x) and 20 (s768i), without central nervous system (CNS) involvement; these mutations are typically resistant to standard therapy. We decided to administer osimertinib because of its favorable toxicity profile and with a view to preventing future CNS relapse.

## Introduction

Adenocarcinoma (ADC) of the lung is the most common pathology related to non-small cell lung cancer (NSCLC). ADC seems to have different precursors, and they are associated with multiple driver mutations. Epidermal growth factor receptor (EGFR) is a receptor located in tumoral cells, which is associated with biological events such as proliferation, motility, invasiveness, resistance to apoptosis, and angiogenesis. These biological events are activated through multiple downstream signaling pathways like the activation of PI3K and RAS as well as interactions with JAK transductor signaling [1].

EGFR incidence in ADC varies based on patients' ethnicity/race; for instance, it is reported to have an incidence of 10% in the white race, 50% among Asians, and 35.8% in Hispanics. The most frequent mutations are deletions in exon 19 and 21 and sensitive mutations. In this report, we present a case of unusual mutations in EGFR in exons 19 and 21 related to resistance to therapy.

## Case presentation

A 65-year-old female presented with a 10-month history of low-back/lumbar pain. She also had a dry cough ongoing for the last six months. Three months after the occurrence of initial symptoms, the patient developed a mass in the right supraclavicular space. The patient was a needlewoman and had a past medical history of hypothyroidism. She was taking levothyroxine, analgesics (hydromorphone and pregabalin), and dalteparin 11500 IU for the thrombosis of the mesenteric vein. Physical exam was positive for an increased anteroposterior diameter of the thorax, decreased bilateral breath sounds, digital hypocratism, and a mass in the supraclavicular space of 3.5 x 3.5 cm, without neurological alterations.

**Figure 1:**
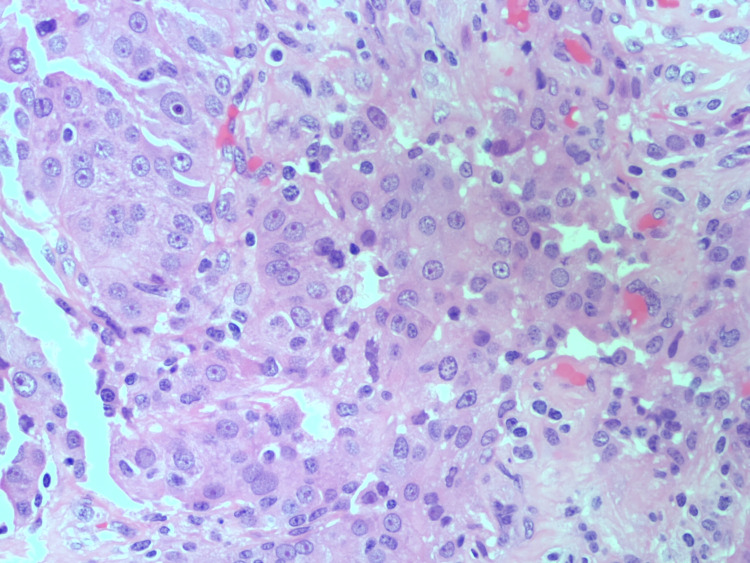
Hematoxylin and eosin stain showing primary pulmonary adenocarcinoma

The chest CT showed a lung mass of 3 x 3 cm in the paratracheal space, with polyostotic compromise and a second mass in the left lower lobe. The abdominal CT showed an unusual thrombosis of the mesenteric vein. A bronchoscopic biopsy of the lung mass performed was consistent with the ADC of the lung. The hematoxylin and eosin stain showed primary pulmonary ADC (Figure 1). Immunohistochemistry study was found to be positive for napsin, thyroid transcription factor-1 (TTF-1), CK7, and AE1/AE3, and negative for p40, CK20, and CD56. Two unusual mutations in the EGFR gene, in exon 18 (g719x) and 20 (s768i), were found.

Initial treatment involved radiation therapy to the mediastinum, due to the imminence of superior vena cava syndrome. However, after an MRI of the brain showed no disease, we decided to initiate treatment with osimertinib. Despite undergoing four cycles with osimertinib, the patient experienced disease progression, complicated by enlarged mass causing compression of vascular structures in the mediastinum. Hence, second-line therapy with pemetrexed and carboplatin was initiated. However, after two cycles, the patient died due to the progression of the disease.

## Discussion

NSCLC is a heterogeneous disease. Molecular techniques are routinely used for the detection of the mutation of the EGFR in the proto-oncogene BRAF, EML4-ALK fusion, and fusions in the oncogene c-ROS (ROS1). The detection of these molecular abnormalities enables the development of therapies targeting metastatic disease, significantly improving the prognoses of these patients [1,2]. Mutations in EGFR are the most common mutations, occurring in nearly 10% of the patients of the white race and nearly 50% of Asian patients [1]. In Latin America, especially among the Hispanic population, 832 patients were tested for mutations in EGFR, revealing 35.8% EGFR mutations, with 47.9% with E19 deletion and 48.3% with the in-frame L858R mutation [3].

Mutations in EGFR that present in the exons 18 to 21 are classified as common and less common. The common mutations account for 80% of the mutations in EGFR, and they comprise the deletion of 19 and the mutation of the exon 21 L858R. The less common mutations consist of the deletion of the 18, G719x, insertion of the 19, exon 20 S768I, exon 21 L861q, insertions in the exon 20, compound mutation, duplication of the kinase domain 18-25, and rearrangements (EGFR-RAD51 and EGFR-PURB). In preclinical studies, the less common mutations of EGFR had a variable sensitivity to tyrosine kinase inhibitors (TKI), considering sensible mutations S768I, L861Q, and G719X, and were resistant to the insertions of the exon 20 [4,5].

Table 1 illustrates the frequency and sensitivity in in-vitro mutations of EGFR to gefitinib/erlotinib, afatinib, and osimertinib. The clinical characteristics of patients with less common mutations did not seem different from patients with common mutations [6,7]. Most of the randomized clinical trials that evaluated TKI therapy in NSCLC included only patients with common mutations due to the low prevalence of less common mutations. Post-hoc analysis has been performed, as well as combined analysis in clinical trial phase II and III reporting the outcomes of TKI therapy in patients with less common mutations. The objective response rate (ORR), progression-free survival (PFS), and overall survival (OS) vary according to the TKI used (showing better responses in clinical outcomes with afatinib and osimertinib) and according to the mutations (Table 2) [7-11]. Long-term outcomes in these patients are weaker than those described in common mutations [6,12-15].

**Table 1:** Frequency and sensitivity in in-vitro mutations of EGFR to gefitinib/erlotinib, afatinib, and osimertinib* *Adapted from [4,5] EGFR: epidermal growth factor receptor; TKI: tyrosine kinase inhibitor

Type of mutation	EGFR mutation	Frequency	Sensitivity to TKI		
			First-generation (gefitinib/erlotinib)	Second-generation (afatinib)	Third-generation (osimertinib)
Common	Exon 19 Del19	44.8%	Sensitive	Sensitive	Sensitive
	Exon 21 L858R	39.8%	Sensitive	Sensitive	Sensitive
Less common	Exon 18 G719X	3.1%	Sensitive	Sensitive	Sensitive
	Exon 19 Ins19	0.6%	Partially sensitive	Sensitive	Sensitive
	Exon 20 S768I	1.1%	Resistant	Partially sensitive	Partially sensitive
	Exon 20 Ins20	5.8%	Resistant	Resistant	Sensitive
	Exon 21 L861Q	0.9%	Partially sensitive	Sensitive	Sensitive

**Table 2:** Prospective studies of TKI in NSCLC patients with less common EGFR mutations* *Adapted from [4] TKI: tyrosine kinase inhibitor; NSCLC: non-small cell lung cancer; EGFR: epidermal growth factor receptor; ORR; objective response rate; mPFS: median progression-free survival; mOS: median overall survival

		First-generation (gefitinib)	Second-generation (afatinib)		Third-generation (osimertinib)
	Estudio	NEJ002 [7]	LUX-Lung 2, 3 y 6 [6]	Combined analysis [8]	KCSG-LU15-09 [9]
Less common mutation	N	5	38	110	36
	ORR	20%	71.1%	60%	50%
	mPFS (months)	2.2	10.7	-	8.2
	mOS (months)	11.9	19.4	-	-
G719X	N	3	18	55	19
	ORR	0%	77.8%	63.4%	53%
	mPFS (months)	1.8	13.8	-	8.2
	mOS (months)	7.9	26.9	-	-
S786I	N	-	8	8	8
	ORR	-	100%	62.5%	38%
	mPFS (months)	-	14.7	-	12.3
	mOS (months)	-	-	-	-
L861Q	N	2	16	47	9
	ORR	50%	56.3%	59.6%	78%
	mPFS (mo)	8.5	8.2	-	15.2
	mOS (mo)	17.3	17.1	-	-

This case report describes a case of a female patient with combined mutations in G719X and S768I in EGFR. Over a quarter of less common EGFR mutations exist as compound mutation or “complex” and have varying responses to TKI therapy. A possible explanation is that only one mutation is not capable of being responsible for tumoral overgrowth but needs another mutation in order to accomplish biological tumorigenesis [16]. Most studies do not report the differences in outcomes between unique less common mutation versus compound less common mutation. In a retrospective study in Taiwan, the patients who received first-generation TKI for compound less common mutations had a better ORR (68.4% vs. 37.8% p=0.011) and PFS (11.9 vs. 6.5 months, p=0.010) than those with unique less common mutations [6].

An analysis evaluating the use of afatinib as first-line therapy in 35 patients with compound less common mutations in EGFR reported that they had a better time to treatment failure (TTF) (14.7 months) and ORR (77.1%) compared to the results observed in common mutations [9]. The use of osimertinib in compound less common mutation is limited [16,17]. The use of osimertinib is preferred in patients with metastatic disease in the central nervous system (CNS) due to this TKI's pharmacodynamic and pharmacokinetic properties.

The U.S. Food and Drug Administration (FDA) has approved afatinib for the standard management of NSCLC metastatic with less common mutations in EGFR (S768I, L861Q y/o G719X). However, we decided to administer osimertinib to our patient because of its favorable toxicity profile and with a view to preventing any future CNS relapse [12,13].

## Conclusions

The use of osimertinib is preferred in patients with NSCLC with metastatic disease and EGFR uncommon mutation, and in special cases like our case despite not having CNS compromise, due to the protection it provides in the CNS owing to its special penetrant features. All evidence of first- and second-generation TKI on EGFR so far have revealed weaker outcomes compared with common mutations, and this supports the use of osimertinib as the first-line treatment in uncommon mutations.
